# Metabolic health effects of the timing of lifestyle behaviours in a combined lifestyle intervention in adults who have obesity or are overweight with complications: protocol for a two-armed, cluster-randomized, pragmatic trial—a TIMED study

**DOI:** 10.1186/s13063-026-09788-z

**Published:** 2026-05-20

**Authors:** Romy Slebe, Renée de Mutsert, Jean-Pierre Després, David J. T. Campbell, Andries Kalsbeek, Patrick Schrauwen, Joline W. J. Beulens, Femke Rutters

**Affiliations:** 1 Department of Epidemiology and Data Science, Amsterdam UMC, Location Vrije Universiteit Amsterdam, Amsterdam, The Netherlands; 2 Amsterdam Public Health, Health Behaviours & Chronic Diseases, Amsterdam, The Netherlands; 3 Department of Clinical Epidemiology, Leiden University Medical Center, Leiden, The Netherlands; 4 Department of Kinesiology, Université Laval and Centre de Recherche Sur Les Soins Et Les Services de Première Ligne, Québec, QC Canada; 5 Department of Medicine, University of Calgary Cumming School of Medicine, Calgary, AB Canada; 6 Department of Community Health Sciences, University of Calgary Cumming School of Medicine, Calgary, AB Canada; 7 Department of Cardiac Sciences, University of Calgary Cumming School of Medicine, Calgary, AB Canada; 8 Department of Endocrinology and Metabolism, Amsterdam UMC, University of Amsterdam, Amsterdam, The Netherlands; 9 Amsterdam Gastroenterology Endocrinology Metabolism, Amsterdam, The Netherlands; 10 Netherlands Institute for Neuroscience (NIN), Institute of the Royal Netherlands Academy of Arts and Sciences (KNAW), Amsterdam, The Netherlands; 11 Institute for Clinical Diabetology, German Diabetes Center, Leibniz Institute for Diabetes Research at Heinrich Heine University Düsseldorf, Düsseldorf, Germany

**Keywords:** Combined lifestyle intervention, Pragmatic trial, Circadian rhythm, Timed lifestyle, Glycaemia, Body mass index

## Abstract

**Background:**

As done in combined lifestyle interventions (CLI), a combination of physical activity, diet, and sleep interventions is effective in reducing body mass index (BMI) and hyperglycaemia. The timing of the behaviours during the day may also affect metabolic health, due to individuals’ circadian rhythms, but the impact of this is not well known. Thus, the inclusion of education on the timing of lifestyle behaviours may be an important addition to CLIs. Therefore, we aim to investigate the metabolic health effect of adding education on timing of lifestyle behaviours to an online CLI among adults who have obesity or are overweight with complications, compared to standard CLI, and routine care.

**Methods:**

In this two-armed, cluster-randomized, superiority, pragmatic trial, at least ten CLI counselling groups will be cluster-randomized to either the standard CLI or the TIMED CLI, with added education on the timing of sleep, physical activity, and dietary intake. Among these clusters, we aim to recruit approximately 100 participants. The primary outcome is self-reported BMI at baseline, and at 3, 9, and 24 months. Secondary outcomes are health-related quality of life (HRQoL) measured using a questionnaire, self-reported waist circumference, fasting glucose and HbA1c measured using at-home finger prick testing at baseline, and at 3, and 9 months. Adherence will be assessed through questionnaires and attendance to the CLI sessions. In addition, an observational control group of 800 participants from a routine care database will be included for comparison. Recruitment started in July 2024.

**Discussion:**

To our knowledge, this is the first pragmatic, cluster-randomized trial that investigates the incorporation of education on circadian timing of lifestyle behaviours in a routine care CLI. The pragmatic design will enable the investigation of the feasibility and metabolic health effect of implementing these timed interventions in a routine care setting.

**Trial registration:**

ISRCTN registry, protocol number ISRCTN14642827. Registered on 7 December 2023, www.isrctn.com/ISRCTN14642827.

**Supplementary Information:**

The online version contains supplementary material available at 10.1186/s13063-026-09788-z.

## Administrative information

Note: the numbers in curly brackets in this protocol refer to SPIRIT checklist item numbers. The order of the items has been modified to group similar items (see http://www.equator-network.org/reporting-guidelines/spirit-2013-statement-defining-standard-protocol-items-for-clinical-trials/).

**Table:** 

Title {1}	Metabolic health effects of the timing of lifestyle behaviours in a combined lifestyle intervention in adults who have obesity or are overweight with complications: Protocol for a two-armed, cluster-randomized, pragmatic trial – A TIMED study
Trial registration {2a and 2b}.	ISRCTN registry, protocol number: ISRCTN14642827
Protocol version {3}	1^st^ of November 2023 Version 1.0
Funding {4}	This project is funded by The Netherlands Organization for Health Research and Development (ZonMw) [459001021], Dutch Diabetes Research Foundation (Diabetes Fonds) [2019.11.101], Canadian Institutes of Health Research (CIHR) [TNC-174963], and Health-Holland [LSHM20107]. This collaborative project is co-financed with PPP-allowance made available by Health-Holland, Topsector Life Sciences & Health, to stimulate public-private partnerships.
Author details {5a}	1.Department of Epidemiology and Data Science, Amsterdam UMC, Location Vrije Universiteit Amsterdam, Amsterdam, The Netherlands 2.Amsterdam Public Health, Health Behaviours & Chronic Diseases, Amsterdam, The Netherlands 3.Department of Clinical Epidemiology, Leiden University Medical Center, Leiden, The Netherlands 4.Department of Kinesiology, Université Laval and Centre de recherche sur les soins et les services de première ligne, Quebec, QC, Canada 5.Department of Medicine, University of Calgary Cumming School of Medicine, Calgary, AB, Canada 6.Department of Community Health Sciences, University of Calgary Cumming School of Medicine, Calgary, AB, Canada 7.Department of Cardiac Sciences, University of Calgary Cumming School of Medicine, Calgary, AB, Canada 8.Department of Endocrinology and Metabolism, Amsterdam UMC, University of Amsterdam, Amsterdam, the Netherlands 9.Amsterdam Gastroenterology Endocrinology Metabolism, Amsterdam, The Netherlands 10.Netherlands Institute for Neuroscience (NIN), Institute of the Royal Netherlands Academy of Arts and Sciences (KNAW), Amsterdam, The Netherlands 11.Institute for Clinical Diabetology, German Diabetes Center, Leibniz Institute for Diabetes Research at Heinrich Heine University Düsseldorf, Düsseldorf, Germany
Name and contact information for the trial sponsor {5b}	R. Slebe. Amsterdam UMC, location Vrije Universiteit, Epidemiology and Data Science. De Boelelaan 1089a, 1081BT Amsterdam, The Netherlands. E-mail: r.slebe@amsterdamumc.nl
Role of sponsor {5c}	As the sponsor the Amsterdam UMC, location VUmc ‘ Stichting VUmc’ is responsible for the study design, data collection, management and analysis and publication of the result. The funders have been involved in the concept of the study design.

## Introduction

### Background and rationale {6a}

The prevalence of overweight and obesity has been steadily rising over the past several decades, increasing the risk of chronic diseases, such as type 2 diabetes, and decreasing health-related quality of life (HRQoL) [1]. Lifestyle behaviours, such as unhealthy dietary patterns, physical inactivity, and poor sleep are associated with increased risk of overweight and obesity [2, 3]. Improving these behaviours can prevent and reduce overweight, obesity, and associated chronic diseases [4–12].

Combined lifestyle interventions (CLIs) provide education and behavioural change techniques (BCTs) to increase physical activity, improve sleep, and improve diet quality. BCTs regularly used in CLIs are based on the taxonomy of behaviour change techniques by Michi et al. [13], and include the main themes of: goal setting and action planning, providing feedback and mentoring, facilitating social support, and shaping knowledge [14]. Interventions that target a combination of different lifestyle factors have shown to be effective to treat people with obesity, prevent type 2 diabetes, and improve HRQoL [15–26]. Therefore, such CLIs have been implemented in Dutch routine care and are reimbursed for adults who have obesity, or are overweight with one of the following comorbid conditions: type 2 diabetes, vascular diseases, sleep apnoea, osteoarthritis, or increased waist circumference [27].

In addition to the quantity and quality of an individual’s physical activity, food intake, and sleep, the timing of these lifestyle behaviours during the day is important for metabolic health [28, 29]. The human body has various circadian rhythms of roughly 24 h, which are generated in the suprachiasmatic nucleus (SCN) in the hypothalamus. The SCN causes physiological and behavioural rhythms, such as rhythms in the body temperature, hormones, glucose metabolism, and cognition. The SCN is mainly entrained by the light-dark cycle, but is also influenced by external ‘Zeitgebers’, such as artificial light, physical activity, and food intake [30]. In the 24/7 nature of modern industrial societies, disruption of sleep-wake rhythms has become increasingly common, due to the rise of shiftwork, time zone transfers, social jetlag, and exposure to artificial light during nighttime hours. These exposures misalign our circadian rhythms, which is associated with increased prevalence of obesity and related diseases [31–38]. For example, shiftwork has been linked to a 1.17 and 1.10 times higher risk of obesity and diabetes, respectively [39, 40]. Changes to the timing of lifestyle behaviours can, however, show potential benefits for glycaemic and metabolic control. Experimental studies on afternoon or evening exercise, for instance, have shown reductions in glucose levels, compared to morning exercise [41, 42]. Additionally, both experimental and observational studies show that preventing circadian misalignment contributes to improved glycaemic and metabolic control. Misalignment can be prevented by omitting shiftwork, irregular sleep, or disrupted sleep [24, 39, 43–51], avoiding (blue) light exposure before bedtime [52, 53], and meal-related practices, such as consistent breakfast consumption, avoidance of nighttime snacking, early meal timing, consuming the majority of daily caloric intake early in the day, and a restricted eating window [36, 54–69].

Nevertheless, these studies on altering the timing of lifestyle behaviours are purely mechanistic animal studies; short-term experimental studies in humans (~ weeks), in highly controlled settings, and with detailed mechanistic outlooks; or observational studies in large cohorts, looking at associations of (proxies of) timing of lifestyle behaviours between people, instead of within people. Additionally, these studies focus on only one of the lifestyle behaviours (i.e. only timing of food intake). Previous studies have shown that the combined effects of addressing different lifestyle behaviours are often greater in the treatment of individuals with overweight or obesity on various metabolic risk factors, compared to one isolated behaviour [21, 70–72]. We therefore hypothesize that this combined effect may also be found when combining specific timed lifestyle interventions.

**Fig. 1:**
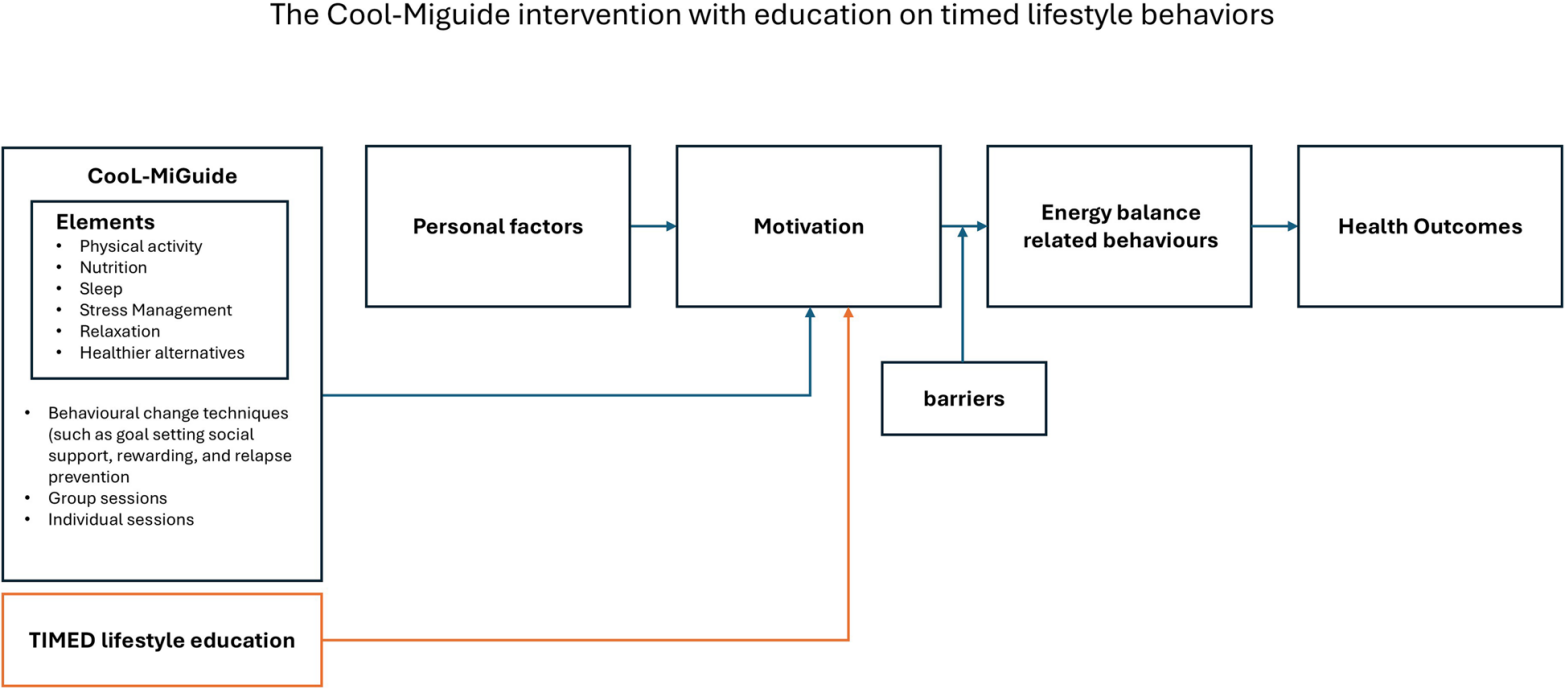
Theoretical framework of the Cool-Miguide intervention with education on timed lifestyle behaviours

In order to test the additional effects of correctly timed lifestyle behaviours in real life, we will add education on timed lifestyle to an existing Dutch CLI. Currently, a widely used CLI in the Netherlands is the ‘Coaching op Leefstijl’ (CooL; translated as Coaching on Lifestyle) intervention. This intervention is a 2-year programme that consists of monthly group sessions and individual sessions. CooL focusses on identifying unhealthy behaviours (concerning physical activity, nutrition, sleep, stress management, and relaxation), and pitfalls, and provides individualized tools to help change towards healthier behaviours [25]. Consequently, participants may prioritize certain behaviours of CooL over behaviours that are less important for the individual, for example one may focus on nutrition while others focus on sleep. Additionally, Miguide translated the CooL intervention in a digital format for participants that cannot join in person sessions. This Cool-MiGuide intervention has the same design, goals, and elements as CooL, but all group and individual sessions are held online. An 8-month pre-post study on CooL-MiGuide showed positive changes in different behaviours (including sedentary time, diet attentiveness, and sleep), resulting in reduced weight and improved perceived health [73]. Previous studies showed that greater improvements in metabolic markers are seen when the same amount of physical activity, sleep, and meal intake is performed aligned with circadian rhythms, compared to misaligned [74, 75]. Therefore, we hypothesize that adding additional education on timed lifestyle behaviours to CooL-MiGuide will also have greater effects, compared to CooL-MiGuide alone. We assume this behaviour change will directly influence circadian alignment by influencing the SCN, which has biological, psychological, and behavioural consequences that we can measure in the form of positive effects on body weight, glycaemic control, fat distribution, lifestyle behaviours, and HRQoL [30]. The theoretical framework of CooL-MiGuide [76], including education on timed lifestyle behaviour, is depicted in Fig. 1. Accordingly, as is customary for the CLI, the interventions (and also the timed education) are based on education and behavioural change techniques, rather than enforced behaviour change. CooL uses various evidence-based techniques and approaches, including BCTs such as goal setting, mobilizing social support, rewarding, and relapse prevention [13, 25]. The effect on metabolic control is therefore dependent on the adherence to the intervention. Therefore, we will directly measure completion of the modules and attendance to the meetings of CooL-MiGuide, adherence to and changes in timed behaviours, as well as metabolic endpoints. With this, we will be able to conceptualize the working mechanisms and potentially be able to better explain our findings.

### Objectives {7}

The objective of this study is to investigate the metabolic health effect of adding education on the timing of lifestyle behaviours to an online CLI, compared to the standard online CLI, and routine care, among adults who have obesity or are overweight with complications. The primary outcome of body mass index (BMI), and secondary outcomes of fasting glucose, HbA1c, HRQoL, and waist circumference will be assessed across four timepoints (baseline, and at 3, 9, and 24 months).

### Trial design {8}

This study is a two-armed, cluster-randomized, superiority, pragmatic trial with two comparator groups. At least 10 groups will be cluster-randomized 1:1 to one of the intervention arms: the standard CLI or the TIMED CLI, with added education on timing of lifestyle behaviours. In addition, an additional observational arm will be used for exploratory analyses to distinguish results attributable to the standard CLI from those reflecting additive effects of the timed education in the TIMED CLI. To keep the trial pragmatic and reduce participant burden, instead of active recruitment of a waiting list control group, 800 participants from the routine care database called the Diabetes Management and Treatment (*DIAMANT*) cohort will be included for exploratory purposes only. These exploratory analyses will enable us to distinguish effects attributable to the standard CLI from those reflecting additive effects of the timed education. This cohort, initially for patients with diabetes, recently expanded with all patients with risk factors of type 2 diabetes: cardiometabolic diseases, overweight or obesity, dementia, and depression. These participants will be matched 8:1 based on age, sex, comorbid condition, and postal codes with participants in the two intervention arms.

## Methods: participants, interventions, and outcomes

### Study setting {9}

Individual and group-based sessions will take place using online video calls, with all information given via an online platform and a mobile app. Data collection was limited to participants living in The Netherlands, as test tubes for HbA1c assay needed to be sent back to the clinical laboratory in Amsterdam, The Netherlands, in a relatively short period of time. Recruitment of this trial started in July 2024, with the treatment period and first data collection starting in September 2024. The entire study will continue over a period of 3 years.

### Eligibility criteria {10}

Eligible study participants are adults with an indication to be referred to a CLI by their general practitioner (Table 1). In addition, participants are excluded if they (1) are unable to provide written informed consent; (2) have serious mental impairment which would prevent them from understanding the study aim; (3) are pregnant; (4) work night shifts; and/or (5) take sulfonylurea medication. The latter is excluded, as the effects of reducing the eating window (part of the TIMED CLI) on the risk of hypoglycaemia in participants taking sulfonylurea medication is not sufficiently known.

**Table 1:** Eligibility criteria for inclusion in the pragmatic trial: Indication for referral to a CLI

• Aged 18 years or older • Either (1) obesity (BMI ≥ 30 kg/m^2^); or (2) overweight (BMI ≥ 25 and < 30 kg/m^2^) with at least one comorbid condition: ◦ Sleep apnoea ◦ Vascular diseases ◦ Type 2 diabetes ◦ Osteoarthritis ◦ Increased waist circumference (men ≥ 102 cm; women ≥ 88 cm) • Motivated to change lifestyle behaviour • Digitally skilled and in the possession of a smartphone • Being able to speak and understand the Dutch language	

*CLI *Combined Lifestyle Intervention, *BMI *body mass index

### Who will take informed consent? {26a}

Informed consent will be obtained via the Electronic Data Capturing software (Castor EDC) [67]. An invite to the electronic informed consent form will be sent by mail after the coordinating researcher talked to the participant to answer any questions and before the start of any study-related assessments.

### Additional consent provisions for collection and use of participant data and biological specimens {26b}

This is not applicable, as no ancillary studies will be performed.

## Interventions

### Explanation for the choice of comparators {6b}

#### Standard CLI

Participants randomized to the standard CLI receive the online CLI (CooL-MiGuide) as it is currently provided as Standard of Care. In short, the programme consists of a basic programme (6–8 months) and a maintenance phase (16–18 months), each including four individual sessions (45 min each) and eight group sessions (90 min each) to help participants change their lifestyle behaviours. All sessions (both individual and group sessions) will be held with the same registered lifestyle coach. Participants can use the MiGuide app and online platform to access more information and to help them adhere to the lifestyle changes. CooL-MiGuide aims to identify individual unhealthy behaviours and pitfalls and provide personalized tools to help change towards healthier behaviours. Consequently, participants may prioritize certain behaviours that are of importance to them. The standard evaluations of MiGuide will provide insight in changes in the quantity of the behaviours throughout the programme and help identify which behaviours they focused on to change. The standard CLI consists of pre-approved topics that are explained in presentations and on the MiGuide platform. All coaches received training by MiGuide in order to provide the CLI in the same way. No alterations are made to this standard CLI. The standard CLI did not include any information about timed lifestyle, and no information was left out. A full overview of the standard CLI can be found in Additional file 1. However, participants have access to external information on timed lifestyle behaviours, such as on social media or news platforms. Coaches in the control arm are instructed to not discuss timing concepts during the CLI sessions, but they are not forbidden to answer questions on timed lifestyle behaviour by participants.

### Intervention description {11a}

#### TIMED CLI

**Fig. 2:**
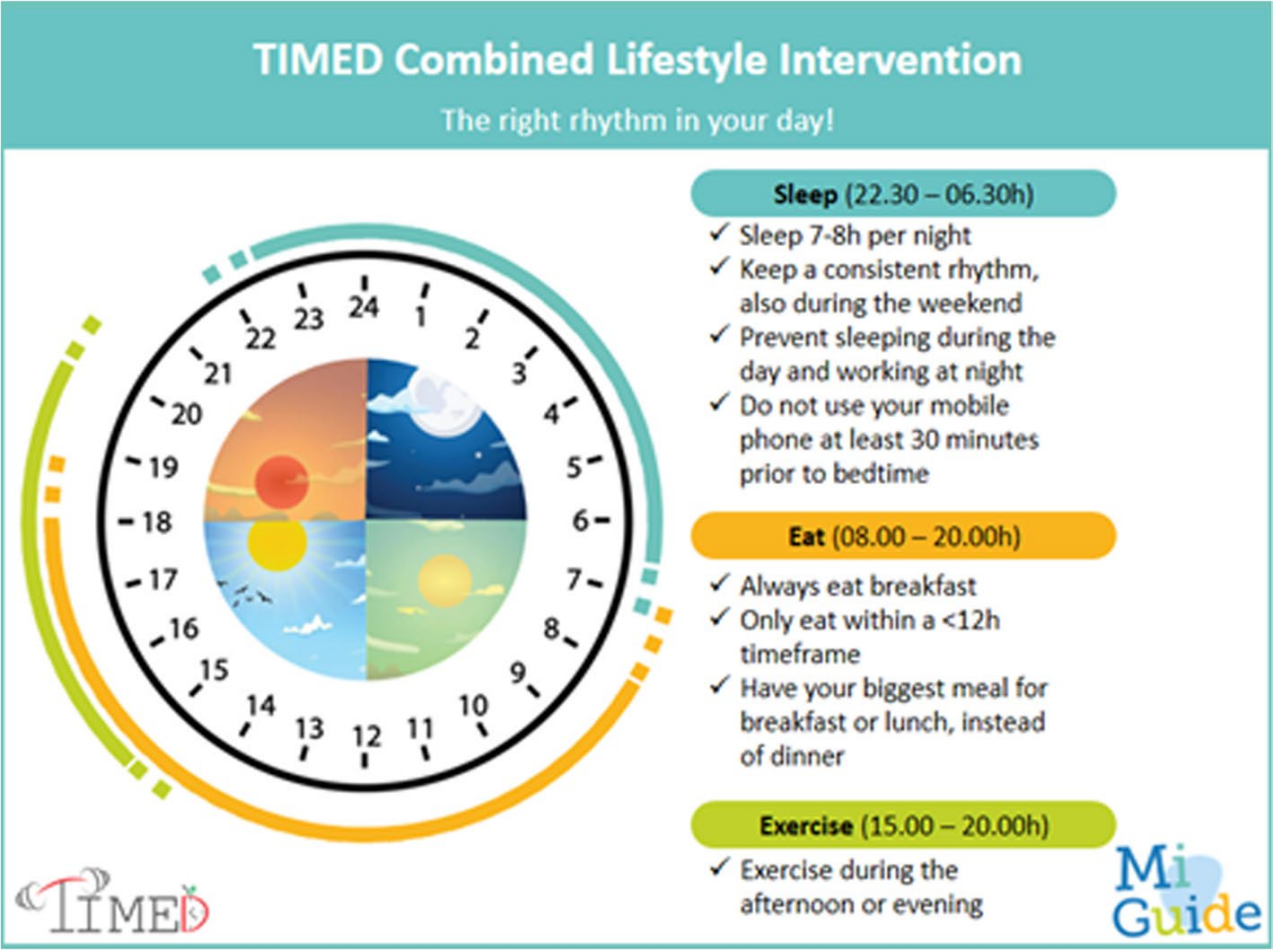
The timed lifestyle advice on sleep, food intake, and exercise for the TIMED GLI

Participants randomized to the TIMED CLI receive the same CLI and can use the app and online platform as described above. In addition, they receive specific education on timing of healthy lifestyle behaviours during the day as depicted in Fig. 2. The education regarding the timing of lifestyle factors is based upon patients’ experiences, systematic reviews of the TIMED consortium [77, 78], and previous observational and experimental research [24, 36, 39, 41–69]. These show that the specific timing of sleep, meal intake, and exercise throughout the day can improve glycaemic and metabolic control. More specifically, the education will consist of the following: (1) sleep 7–8 h per night, with a consistent rhythm on weekdays and weekend days (e.g. between 22.30 and 06.30 h), prevent daytime sleeping and prevent blue light exposure 30 min prior to bedtime; (2) eat within a 12-h eating window (e.g. between 08.00 and 20.00 h), always eat breakfast, and eat the largest part of the daily caloric intake at breakfast or lunch; and (3) schedule exercise in the afternoon or evening (e.g. between 15.00 and 20.00 h). Additional information on the working mechanisms in the human body and practical tips will be explained and repeated in all group and individual sessions, and on the online MiGuide platform.

To minimize the risk of contamination between study arms, separate presentations and distinct sections of the MiGuide platform will be used for each intervention condition. Whether participants access the appropriate content (TIMED CLI or standard CLI platform) and receive the correct presentation will be monitored. Prior to the start of each group, the researcher will confirm the correct group allocation with the lifestyle coach. In addition, one of the researchers attends the first group session to provide additional information about the research and verifies that the participants receive the presentation corresponding to the allocated study arm. As mentioned, participants identify their individual unhealthy behaviours and pitfalls, and may therefore focus on personalized selection of behaviour(s). Although all timed recommendations are explained as equally important during the sessions, participants may prioritize certain recommendations based on their personal preferences and problems. Changes in the quantity and the timing of different lifestyle behaviours will be monitored, as described in the Outcomes section.

### Criteria for discontinuing or modifying allocated interventions {11b}

There are no criteria of discontinuing or modifying the allocated intervention. Since the trial is pragmatic, participants receive additional education, and discuss regularly with the assigned lifestyle coach which factors they will work on. This is part of the working mechanisms of the standard CLI.

### Strategies to improve adherence to interventions {11c}

There are several strategies in place to improve adherence to the intervention. First, there will be a presentation for all groups as part of the first group session by the researcher, which participants must attend. In this presentation, information regarding the research will be discussed. Additionally, participants of the TIMED CLI will receive education on the working mechanisms and advice during this presentation, and this education will be reiterated during other group sessions and individual sessions by the lifestyle coach. All information is also available on the MiGuide online platform, which is accessible at all times and will be stimulated by the coaches.

### Relevant concomitant care permitted or prohibited during the trial {11d}

There are no restrictions regarding concomitant care and medication use during this study.

### Provisions for post-trial care {30}

After the trial, participants from both groups will be instructed to keep up their new lifestyle behaviours, including the timed lifestyle behaviours within the TIMED CLI. After 2 years, these behaviours are expected to have been incorporated into the participants’ routine.

### Outcomes {12}

#### Primary outcomes

##### BMI

BMI is selected as the primary outcome of this study, because weight reduction is the main aim of the CLI. Given the pragmatic nature of our study design, we aim to maximize the use of the existing intervention while minimizing the need for additional modifications. As BMI is routinely assessed within the standard evaluations all CLIs, its use aligns with the pragmatic approach and limits additional burden to the participant. In addition, we prioritized BMI over other metabolic outcomes, as BMI represents changes in energy balance, it is most reported in other studies on CLI and/or timing of lifestyle, which enhances comparability of our results, as well as being an easy-to-measure outcome, which would aid implementation of the intervention. Participants measure their bodyweight in the morning with an empty bladder, wearing only their underwear, and their height while barefoot, at baseline, and at 3, 9, and 24 months. BMI will be calculated by dividing bodyweight in kilograms by height in meters squared (kg/m^2^).

### Secondary outcomes

#### Fasting blood glucose and HbA1c

Participants receive the Contour Plus Blue glucose monitor, including test strips, lancing devices, and an information sheet. Using the device, they measure their blood glucose in the morning after an overnight fast (no food and drinks for ≥ 10 h) with a fingertip prick, at baseline, and at 3, and 9 months. The result, together with a photo of the measurement on the device, will have to be entered in the electronic questionnaire sent by email. The Contour Plus Blue glucose monitor was tested in different studies, showing compliance with the International Organization for Standardization accuracy criteria (ISO 15197:2013 [79]) and significantly lowers mean absolute relative differences from the reference value, compared to four other blood glucose monitor systems [80, 81].

HbA1c levels will be estimated using a high-performance liquid chromatography assay, at baseline, and at 3, and 9 months. Participants will collect four drops of blood from a fingertip prick in an HbA1c test tube using the materials and instructions provided. The test tubes will be sent to the Amsterdam UMC – VUmc clinical laboratory to perform the assay, using a medical return envelope, at the day of collection. When temperatures are < 4 or > 25 °C, participants will be asked to drop off the package at a parcel delivery point. This procedure was previously tested and showed feasible and reliable results, with an uncertainty of 3.7% [82].

#### Waist circumference

Participants will receive a measuring tape and instructions to measure their waist circumference in centimetres at the level midway between the lowest rib margin and the iliac crest while standing in a relaxed standing position with the feet approximately 30 cm apart. Waist circumference will be collected at baseline, and at 3, 9, and 24 months.

#### Health-related quality of life

The Dutch form of the PROMIS Scale v1.2 – Global Health will be used to assess mental and physical health. The scale is efficient in the assessment of self-reported health and predictive of health care use [83, 84] and will be taken at baseline, and at 3, and 9 months.

#### Timing of food intake

The timing of food intake will be assessed by the chrono-nutrition questionnaire at baseline, and at 3, and 9 months. The questionnaire consists of eight closed questions on meal regularity, seven closed questions on meal frequency, and 11 closed questions on the timing of meals. The questionnaire was first developed for a study on chrono-nutrition and diet quality in adolescents with delayed sleep-wake phase disorder [85]. Questions are based on questionnaires and literature from other studies about the relationships between chrono-nutrition, metabolic syndrome, and the biological clock [85]. This specific timing of eating questionnaire has not been validated; however, similar chrono-nutrition questionnaires show great validity [86].

#### Timing of sleep

A questionnaire consisting of 14 items on sleep duration, insomnia, shiftwork, daytime napping, and use of sleep medication will be taken at baseline, and at 3, and 9 months. The questionnaire is a combination of (parts of) the validated Munich Questionnaire and the validated Epworth Sleepiness Scale [87–89].

#### Timing of physical activity

No golden standard regarding questions on timing of physical activity exists. Thus, separate questions will be asked regarding the timing of physical activity at baseline, and at 3, and 9 months.

#### Acceptability of the intervention

To measure the acceptability of the intervention, the generic form of the validated theoretical framework of acceptability questionnaire [90] will be used, which was translated to Dutch. Subdomains of the acceptability questionnaire will be used to evaluate the affective attitude, perceived burden, perceived effectiveness, and self-efficacy regarding the intervention. This questionnaire will be taken at baseline, and at 3, and 9 months.

#### General questionnaires

The general questionnaires used in the MiGuide evaluation include questions on education level, employment status, alcohol consumption, smoking behaviour, physical activity, and dietary intake through a food diary. This questionnaire will be taken at baseline, and at 3, 9, and 24 months.

#### Participant timeline {13}

Participants will be informed about the study through a participant information folder and informed consent form. Participants will be asked to provide consent for the use of their data gathered via the MiGuide app and platform, as well as through the evaluation forms used by MiGuide.

To evaluate the effects of the interventions, the above-mentioned outcomes will be measured at different times during the study (see Table 2 for an overview of measurements). A part of this data is routinely collected as part of standard care; however, some additional measurements are included specifically for this study. All measurements and questionnaires are asked in participants in the standard CLI and TIMED CLI.

**Table 2:**
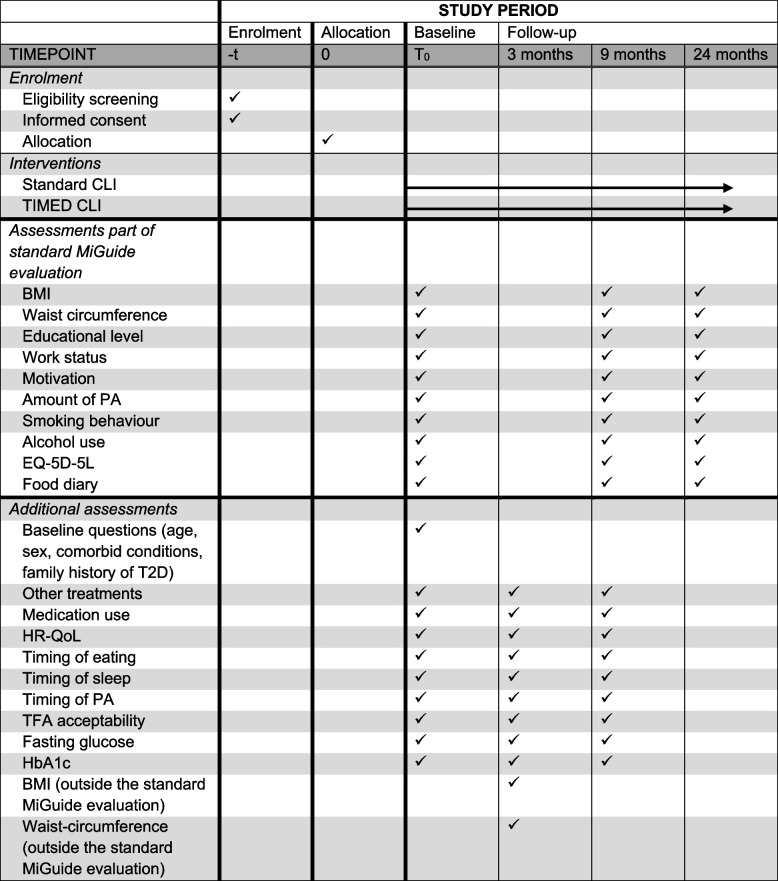
Schedule of enrolment, interventions, and assessments of the pragmatic trial

*BMI *body mass index, *PA *physical activity, *EQ-5D-5L *Euro-Quality of Life 5-dimensional questionnaire, *T2D *type 2 diabetes, *HR-QoL *health-related quality of life, *TFA *theoretical framework of acceptability

### Sample size {14}

To our knowledge, no previous study has compared effects of education on timing of a combination of different lifestyle behaviours, with routine care CLI on any outcome. We could thus not use such studies to inform our power calculation. Therefore, we based our sample size calculation on our primary outcome of BMI based on a study comparing caloric restriction with and without time-restricted eating in participants with obesity [91]. This study by Liu et al. [91] found a 0.7 kg/m^2^ greater reduction in BMI in time-restricted eating after 12 months compared to caloric restriction alone. Although cluster randomization will be used, we expect minimal within-cluster correlation, since the clustering is solely on participants’ personal schedules rather than shared characteristics such as place of residence. To further minimize potential correlation, groups will be scheduled on different days and at different times of day. To detect a difference of 0.7 ± 0.9 kg/m^2^ in BMI with an *α* of 0.05, and *β* of 0.1, an intraclass correlation coefficient of 0.02, and a cluster size of 8–12 participants, we calculated that 45 participants per group are required. Accounting for 10% attrition rate, a total of 50 participants per group will be enrolled.

### Recruitment {15}

Recruitment for the CLI groups will be conducted through four networks: (1) general practitioners of Extramural Leiden University Medical Center Academic Network; (2) general practitioners of ‘Huisartsenorganisatie West-Friesland’; (3) the Diabetes Care System [92]; and (4) CooL-MiGuide. If necessary, additional recruitment will be carried out through other general practitioners or via social media.

## Assignment of interventions: allocation

### Sequence generation {16a}

A minimum of ten groups will be scheduled for the pragmatic trial. These groups will be cluster-randomized (1:1) to either the TIMED CLI or the standard CLI using a randomization list (block size of 4) made by an independent statistician using *Randomizr* packages (version 1.0.0) in RStudio (version 4.4.0).

### Concealment mechanism {16b}

Randomization of each group will be performed by the coordinating researcher after the enrolment of that group is completed. During the enrolment period and at the time of group allocation, both the participants and scheduling staff will remain unaware of the assigned intervention, as randomization has not been performed at that point.

### Implementation {16c}

Prior to randomization, participants are assigned to a group based on their personal scheduling preferences for time and day by scheduling staff.

## Assignment of interventions: blinding

### Who will be blinded {17a}

Blinding of both participants and lifestyle coaches is not feasible due to the study design, as both are aware of whether additional education on timing of lifestyle factors is provided. To minimize bias, endpoint evaluation will be performed by an independent statistician.

### Procedure for unblinding if needed {17b}

Since the research team and participants are not blinded to the treatment allocation, unblinding procedures are not needed.

## Data collection and management

### Plans for assessment and collection of outcomes {18a}

Participants will receive a home kit to measure their fasting glucose and HbA1c. HbA1c levels will be assessed using a high-performance liquid chromatography assay at the Amsterdam UMC – VUmc clinical laboratory, and fasting glucose will be measured using the Contour Plus Blue glucose monitor. Lab results will be entered in the electronic case report forms (eCRF) in Castor EDC as soon as they are determined by the laboratory. Questionnaires will be completed by the participants using Castor EDC. Data extracted from the MiGuide platform, including anthropometric measures, will also be incorporated.

### Plans to promote participant retention and complete follow-up {18b}

Since we are recruiting participants that have already committed to joining a CLI, we anticipate that these individuals will be committed to the CLI. To promote participant retention and ensure complete follow-up, the research burden has been intentionally minimized, reflecting the pragmatic nature of the trial. Participants will receive all data collection materials at home, including self-addressed return envelopes, and will complete a limited number of online questionnaires from home. Reminder emails will be sent at each data collection time point, and participants who are unable to fingerpick themselves may request assistance from the research team or their local general practitioner.

### Data management {19}

We will use Castor EDC as a clinical data management system to capture all collected data in eCRFs [93]. Data validation checks and notifications are embedded in the eCRF to ensure correct data entry.

### Confidentiality {27}

The handling of personal data complies with the Dutch Personal Data Protection Act (AVG/GDPR). The data of all participants that will participate in the present study will be coded. All participants recruited from the Diabetes Care System have an identification number, consisting of five numbers, obtained during previous studies. All other participants will receive a new identification number. These numbers cannot be traced to the person without the key, which is stored separately. The key document is only available to the participating investigators. Participants can request the information stored in their eCRF.

### Plans for collection, laboratory evaluation and storage of biological specimens for genetic or molecular analysis in this trial/future use {33}

Biological samples collected during this study will be analyzed during the study. All remaining materials will be destroyed afterwards.

## Statistical methods

### Statistical methods for primary and secondary outcomes {20a}

Baseline characteristics will be described by intervention arm as mean ± standard deviation, median and interquartile range (if not normally distributed), or numbers and percentages if categorical. Statistical analyses will be performed using RStudio. An intention-to-treat analysis will be performed for both primary and secondary outcomes using multiple linear mixed models to compare changes between baseline, and at 3, and 9 months and compare between intervention arms. For BMI and waist circumference, effects will also be estimated at 24 months. The models will account for clustering within CLI groups, and outcomes that are not normally distributed will be log-transformed prior to analysis.

### Interim analyses {21b}

Interim analyses will not be performed for this study.

### Methods for additional analyses (e.g. subgroup analyses) {20b}

Additional exploratory analyses will be performed to get a better understanding of the effectiveness of the intervention in certain subgroups. Therefore, analyses will be stratified to evaluate whether certain subgroups (e.g., early versus late chronotype, participants with versus without type 2 diabetes) benefit more from the intervention, compared to others. In addition, a greater shift towards desired timing behaviours will be associated with metabolic improvements, to see whether a dose–response relationship can be found. Furthermore, acceptability of the added education will be assessed by comparing the acceptability questionnaire between the standard CLI and TIMED CLI. In addition, the results of the standard CLI and TIMED CLI will be compared with an additional arm from the general care database DIAMANT. It will enable us to distinguish effects attributable to the standard CLI from those reflecting additive effects of the timed education. This is only for exploratory purposes and will not be used to draw any causal conclusions on the effect of the standard CLI itself.

### Methods in analysis to handle protocol non-adherence and any statistical methods to handle missing data {20c}

Compliance to the standard CLI or TIMED CLI will be tested based on the percentage of completion of modules on the MiGuide platform and attendance to the group meetings. Both completion of ≥ 80% of the modules, as well as ≥ 80% of the sessions, will be defined as compliant [94]. Both intention-to-treat and per-protocol analyses will be performed based on this compliance to the CLI. Additionally, adherence to the timed lifestyle behaviours will be self-reported and based on the timing of eating questionnaire, sleep questionnaire, and timing of physical activity questionnaire. These questionnaires will provide insight in adherence to the timed recommendations for all participants, both at baseline as well as changes during the follow-up period. To assess adherence, several sensitivity analyses will be conducted, comparing the timing of a certain lifestyle behaviour between baseline and follow-up. For example, participants who are already engaging in physical activity predominantly in the afternoon or evening, or those who transition towards physical activity in the afternoon or evening over the course of the study, will be considered adherent to the physical activity timing recommendations. This approach enables us to capture partial adherence to the different behaviours.

Fidelity of the coaches is assessed by checking the presentations that were given by the coaches during the session, which are uploaded to the MiGuide platform for the participants. When sessions are cancelled (due to unforeseen circumstances) and not rescheduled, this will be noted in a deviation form protocol by the researchers. Contamination of the timed advices between the TIMED CLI and standard CLI will be assessed by comparing the compliance results and adherence results. Furthermore, mixed model analysis will be used, which is able to account for missingness. We will therefore not do any imputation for missing data. Imbalances in participants’ characteristics between groups will not be tested statistically. However, clinically relevant imbalances will be addressed through sensitivity analyses that adjust for these variables.

### Plans to give access to the full protocol, participant level-data and statistical code {31c}

After finalization and publication of the study, access to the protocol and statistical code can be requested.

## Oversight and monitoring

### Composition of the coordinating centre and trial steering committee {5d}

Dr. F. Rutters is the primary investigator of this study and responsible for ethical approval, coordination, and execution of the trial. MSc R. Slebe is the coordinating researcher, who is responsible for data collection and data management. As this is a pragmatic trial, there is no trial steering committee in place.

### Composition of the data monitoring committee, its role and reporting structure {21a}

There is no data safety monitoring board or safety committee established for this study given its low-risk profile.

### Adverse event reporting and harms {22}

To reduce the chance of adverse events (i.e. risk of hypoglycaemia), people taking sulfonylurea medication will be excluded from participation. Second, possible adverse events will be explained in the first group session and participants are advised to discuss possible events with their lifestyle coach and general practitioner. Given the non-invasive nature of the study procedures and intervention, no serious adverse events are anticipated. Any events that are spontaneously reported by participants or observed by the investigator and that have a sustained effect on the physical wellbeing of the participant will be documented and reported.

### Frequency and plans for auditing trial conduct {23}

The study site will not be monitored by an independent monitor of the Amsterdam UMC. Instead, an independent colleague will review the study files, the original source documents, and the eCRFs to verify accuracy of data collection and processing.

### Plans for communicating important protocol amendments to relevant parties (e.g. trial participants, ethical committees) {25}

Substantial changes to the protocol will be submitted for review to the ethical committee in an amendment. If applicable, the participant information will be adjusted accordingly. Additionally, study forms, operating manuals and the trial registration will be updated. The current version of the study protocol (version 1.0, 1 November 2023) is approved on 9 January 2024.

### Dissemination plans {31a}

We aim to publish the study results in an international, peer-reviewed, medical journal. Further dissemination will be discussed with patient partners within the TIMED consortium. Dissemination plans include making a podcast, as well as e-learnings on different platforms to reach different audiences.

## Discussion

To date, most research has focused on animal models or short-term experiments in humans (ranging from 24 h to 3 weeks) that examined often single lifestyle factors. Thus, it remains unclear whether improvements in metabolic control can be achieved under real-life by modifying the timing of daily behaviours. Our pragmatic trial will provide insights into whether adding relatively simple education on the timing of lifestyle factors to a CLI can have an additive effect on body weight, glycaemic control, fat distribution, lifestyle behaviours, and quality of life. The pragmatic nature of our study setting enables us to reflect the effect of timed behaviour in real-world conditions, close to routine care. This allows us for an accurate understanding of how these interventions perform in routine care, as opposed to highly controlled experimental settings. This approach offers valuable insights into the feasibility and effectiveness within routine care, enhancing the external validity of the findings. In contrast to current evidence, which mainly relies on highly controlled settings on individual lifestyle factors, our study ensures that results are applicable in real-world settings. Nevertheless, this study design also brings some limitations and points of discussion that should be acknowledged.

First, we include an additional observational arm extracted from the general care database *DIAMANT*. This arm is used for exploratory analyses and can therefore not be used to draw causal conclusions. The arm might inform us on results attributable to the standard CLI from those reflecting additive effects of the timed education. This is an ethical alternative to an additional waiting list control, which maintains the pragmatic nature of the trial and reduces participant burden. However, this database is a non-randomized comparator and has slightly different assessments (i.e. other assays to measure HbA1c) and measuring time points (e.g. one month earlier or later), compared to our study design. We will use matching procedures (1:8 ratio) to minimize baseline imbalances based on age, sex, comorbid conditions, and postal codes, and include participants that have at least two measurement moments over a period of nine months. Nevertheless, residual confounding is always of risk and these comparisons with the observational database should always be interpreted with caution.

Second, due to the fully online nature of the intervention and budget limitations, objective in-person measures are not feasible. We feel the self-reported methods are most suitable with the pragmatic design of the study and seem sufficient to answer the research question. In a previous study [95], 99% and 94% of the participants successfully measured their waist circumference and completed their HbA1c test, respectively. Only 22% of the participants failed the first attempt of blood measurements, but a second attempt was often successful. In case of difficulties, participants can request help from the research staff. Furthermore, a systematic review [96] on the validity of self-reported weight and height reported good agreement between measured and self-reported weight and height, making it specifically feasible in larger and online epidemiological investigations. However, factors such as age, sex, and social economic position may influence the underestimation or overestimation of weight and height, and should be taken into account when interpreting the results, especially when participants are classified into risk groups based on BMI. To minimize the risk of bias, participants will receive extensive written instructions at each measuring occasion, and all measurement procedures will be explained by the research staff during the first group session.

Third, we anticipate some influence of the Hawthorne effect (the tendency to alter behaviour due to the awareness of being observed), as well as social desirability bias in all participants. We expect that social desirability bias may be more pronounced in participants of the TIMED CLI, as they are aware of the added education on timing behaviours, and know the desirable timing of meal intake, exercise, and sleep. This could overestimate the compliance to these behaviours in participants of the TIMED CLI, compared to participants of the standard CLI. Within-person changes over time might therefore be more reliable than between-person comparisons. These will therefore be explored when analyzing the results.

Fourth, the primary outcome of BMI might be a conservative endpoint, as it reflects multiple underlying biological, psychological, and behavioural mechanisms that are also influenced by circadian rhythms. Consequently, changes in these mechanisms may not translate into detectable changes in BMI, but still represent important and clinically relevant changes and effects, for example reductions in energy intake or improvements in sleep. We therefore try to capture these mechanisms in the secondary outcomes. Additionally, null findings may be attributable to deviations in intervention delivery, lack of clarity in instructions, or insufficient explanation of the timed advice. To enhance the quality and consistency of intervention delivery, regular meetings with lifestyle coaches and standardized presentations are used. Compliance, adherence and perceived barriers are monitored through questionnaires, completion of the modules, and attendance to the group meetings. However, to provide a full understanding of the perceived barriers to adopt each timing recommendation, a dedicated feasibility study will be needed.

Finally, our results will help determine whether education on timed lifestyle are of added value to the CLI, which could also easily be generalizable to other CLIs in the Netherlands. However, the design also limits some of its generalizability. First, as shift workers are excluded from our study, the results will only be generalizable to individuals with a relatively stable circadian schedule. Shift workers are a well-known high-risk population with an unstable circadian rhythm that could specifically benefit from interventions on the timing of lifestyle behaviours. However, to meet the needs of this specific population, the intervention would need to be adopted with specific advice. As currently, advice like regular sleep during the night is not feasible with shift work. A specifically designed intervention for shift workers would possibly have even larger effects on health outcomes, compared to this current relative stable population, due to their misaligned state. Second, the digital literacy requirements may result in a younger study population with higher social economic position, compared to participants of other, offline CLIs. Last, given the Dutch healthcare context, in which CLIs are already reimbursed, results may not be easily transferred to other non-Dutch settings.

Nevertheless, education about timing is a low-cost and easy enhancement to the existing CLIs, as long as there is an infrastructure present to share the presentations, like the MiGuide platform. If found effective, education on timing can be seen as an addition to existing CLIs, as well as to routine care. Even null findings will provide us with valuable information, and give us the opportunity to research why effects found in highly controlled settings may not be transferable to real-life settings.

### Trial status

The current trial status is ongoing. The study started in July 2024, and recruitment was completed in July 2025. The treatment period and first data collection started in September 2024. The last data collection for the 9-month follow-up is expected to be in April 2026. Around July 2027, the collection of the 24-month follow-up from the MiGuide evaluation will be completed.

## Supplementary Information

Additional file 1.

## Data Availability

The datasets used and/or analyzed during the current study are available from the corresponding author upon reasonable request.

## Abbreviations

BCT: Behavioural Change Techniques
BMI: Body Mass Index
CLI: Combined Lifestyle Intervention
CooL: Coaching op Leefstijl [Dutch for Coaching on Lifestyle]
DIAMANT: The Diabetes Management and Treatment cohort
eCRFs: Electronic Case Report Forms
HRQoL: Health-Related Quality of Life
SCN: Suprachiasmatic Nucleus

## References

[1] Kolotkin RL, Andersen JR. A systematic review of reviews: exploring the relationship between obesity, weight loss and health-related quality of life. Clin Obes. 2017;7(5):273–89. 28695722 10.1111/cob.12203 PMC5600094

[2] Kopp W. How Western diet and lifestyle drive the pandemic of obesity and civilization diseases. Diabetes Metab Syndr Obes. 2019;12:2221–36. 31695465 10.2147/DMSO.S216791 PMC6817492

[3] Silveira EA, Mendonca CR, Delpino FM, Elias Souza GV, Pereira de Souza Rosa L, de Oliveira C, et al. Sedentary behavior, physical inactivity, abdominal obesity and obesity in adults and older adults: a systematic review and meta-analysis. Clin Nutr ESPEN. 2022;50:63–73. 10.1016/j.clnesp.2022.06.001 35871953

[4] Ajala O, English P, Pinkney J. Systematic review and meta-analysis of different dietary approaches to the management of type 2 diabetes. Am J Clin Nutr. 2013;97(3):505–16. 23364002 10.3945/ajcn.112.042457

[5] Meng Y, Bai H, Wang S, Li Z, Wang Q, Chen L. Efficacy of low carbohydrate diet for type 2 diabetes mellitus management: a systematic review and meta-analysis of randomized controlled trials. Diabetes Res Clin Pract. 2017;131:124–31. 28750216 10.1016/j.diabres.2017.07.006

[6] Garcia-Molina L, Lewis-Mikhael AM, Riquelme-Gallego B, Cano-Ibanez N, Oliveras-Lopez MJ, Bueno-Cavanillas A. Improving type 2 diabetes mellitus glycaemic control through lifestyle modification implementing diet intervention: a systematic review and meta-analysis. Eur J Nutr. 2020;59(4):1313–28. 31781857 10.1007/s00394-019-02147-6

[7] Esposito K, Maiorino MI, Bellastella G, Chiodini P, Panagiotakos D, Giugliano D. A journey into a Mediterranean diet and type 2 diabetes: a systematic review with meta-analyses. BMJ Open. 2015;5(8):e008222. 26260349 10.1136/bmjopen-2015-008222 PMC4538272

[8] Pan B, Ge L, Xun YQ, Chen YJ, Gao CY, Han X, et al. Exercise training modalities in patients with type 2 diabetes mellitus: a systematic review and network meta-analysis. Int J Behav Nutr Phys Act. 2018;15(1):72. 30045740 10.1186/s12966-018-0703-3 PMC6060544

[9] Zhao X, He Q, Zeng Y, Cheng L. Effectiveness of combined exercise in people with type 2 diabetes and concurrent overweight/obesity: a systematic review and meta-analysis. BMJ Open. 2021;11(10):e046252. 34615674 10.1136/bmjopen-2020-046252 PMC8496382

[10] Mannucci E, Bonifazi A, Monami M. Comparison between different types of exercise training in patients with type 2 diabetes mellitus: a systematic review and network metanalysis of randomized controlled trials. Nutr Metab Cardiovasc Dis. 2021;31(7):1985–92. 33965297 10.1016/j.numecd.2021.02.030

[11] Picard M, Tauveron I, Magdasy S, Benichou T, Bagheri R, Ugbolue UC, et al. Effect of exercise training on heart rate variability in type 2 diabetes mellitus patients: a systematic review and meta-analysis. PLoS One. 2021;16(5):e0251863. 33999947 10.1371/journal.pone.0251863 PMC8128270

[12] Jing X, Chen J, Dong Y, Han D, Zhao H, Wang X, et al. Related factors of quality of life of type 2 diabetes patients: a systematic review and meta-analysis. Health Qual Life Outcomes. 2018;16(1):189. 30231882 10.1186/s12955-018-1021-9 PMC6147036

[13] Michie S, Richardson M, Johnston M, Abraham C, Francis J, Hardeman W, et al. The behavior change technique taxonomy (v1) of 93 hierarchically clustered techniques: building an international consensus for the reporting of behavior change interventions. Ann Behav Med. 2013;46(1):81–95. 23512568 10.1007/s12160-013-9486-6

[14] Bossen D, Bak M, Braam K, Wentink M, Holla J, Visser B, et al. Online and offline behavior change techniques to promote a healthy lifestyle: a qualitative study. Int J Environ Res Public Health. 2022;19(1):521. 10.3390/ijerph19010521 PMC8744993 35010781

[15] Barreira E, Novo A, Vaz JA, Pereira AMG. Dietary program and physical activity impact on biochemical markers in patients with type 2 diabetes: a systematic review. Aten Primaria. 2018;50(10):590–610. 29061310 10.1016/j.aprim.2017.06.012 PMC6836882

[16] Cradock KA, G OL, Finucane FM, Gainforth HL, Quinlan LR, Ginis KA. Behaviour change techniques targeting both diet and physical activity in type 2 diabetes: a systematic review and meta-analysis. Int J Behav Nutr Phys Act. 2017;14(1):18. 28178985 10.1186/s12966-016-0436-0 PMC5299734

[17] Chen L, Pei JH, Kuang J, Chen HM, Chen Z, Li ZW, et al. Effect of lifestyle intervention in patients with type 2 diabetes: a meta-analysis. Metabolism. 2015;64(2):338–47. 25467842 10.1016/j.metabol.2014.10.018

[18] Gillies CL, Abrams KR, Lambert PC, Cooper NJ, Sutton AJ, Hsu RT, et al. Pharmacological and lifestyle interventions to prevent or delay type 2 diabetes in people with impaired glucose tolerance: systematic review and meta-analysis. BMJ. 2007;334(7588):299. 17237299 10.1136/bmj.39063.689375.55 PMC1796695

[19] Balk EM, Earley A, Raman G, Avendano EA, Pittas AG, Remington PL. Combined diet and physical activity promotion programs to prevent type 2 diabetes among persons at increased risk: a systematic review for the Community Preventive Services Task Force. Ann Intern Med. 2015;163(6):437–51. 26167912 10.7326/M15-0452 PMC4692590

[20] Schwingshackl L, Dias S, Hoffmann G. Impact of long-term lifestyle programmes on weight loss and cardiovascular risk factors in overweight/obese participants: a systematic review and network meta-analysis. Syst Rev. 2014;3:130. 25358395 10.1186/2046-4053-3-130 PMC4227972

[21] Johns DJ, Hartmann-Boyce J, Jebb SA, Aveyard P, Behavioural Weight Management Review G. Diet or exercise interventions vs combined behavioral weight management programs: a systematic review and meta-analysis of direct comparisons. J Acad Nutr Diet. 2014;114(10):1557–68. 25257365 10.1016/j.jand.2014.07.005 PMC4180002

[22] Collins KA, Ross LM, Piner LW, Fos LB, Slentz CA, Bateman LA, et al. Amount and intensity effects of exercise training alone versus a combined diet and exercise lifestyle intervention on health-related quality of life in the STRRIDE-PD randomized trial. BMJ Open Diabetes Res Care. 2022;10(1):e002584. 10.1136/bmjdrc-2021-002584 PMC8796224 35086944

[23] Marcos-Delgado A, Hernandez-Segura N, Fernandez-Villa T, Molina AJ, Martin V. The effect of lifestyle intervention on health-related quality of life in adults with metabolic syndrome: a meta-analysis. Int J Environ Res Public Health. 2021. 10.3390/ijerph18030887. 33498570 10.3390/ijerph18030887 PMC7908372

[24] Beccuti G, Pannain S. Sleep and obesity. Curr Opin Clin Nutr Metab Care. 2011;14(4):402–12. 21659802 10.1097/MCO.0b013e3283479109 PMC3632337

[25] van Rinsum C, Gerards S, Rutten G, Philippens N, Janssen E, Winkens B, et al. The Coaching on Lifestyle (CooL) intervention for overweight and obesity: a longitudinal study into participants' lifestyle changes. Int J Environ Res Public Health. 2018;15(4):680. 10.3390/ijerph15040680 PMC5923722 29617337

[26] Duijzer G, Haveman-Nies A, Jansen SC, Beek JT, van Bruggen R, Willink MGJ, et al. Effect and maintenance of the SLIMMER diabetes prevention lifestyle intervention in Dutch primary healthcare: a randomised controlled trial. Nutr Diabetes. 2017;7(5):e268. 28481335 10.1038/nutd.2017.21 PMC5518803

[27] Oosterhoff M, Feenstra T, de Wit A. Monitor Gecombineerde Leefstijlinterventie 2023. Bilthoven: Rijksinstituut voor Volksgezondheid en Milieu (RIVM); 2023. Available from: https://www.rivm.nl/sites/default/files/2023-05/Monitor%20Gecombineerde%20Leefstijlinterventie%202023_TG.pdf.

[28] Stenvers DJ, Scheer F, Schrauwen P, la Fleur SE, Kalsbeek A. Circadian clocks and insulin resistance. Nat Rev Endocrinol. 2019;15(2):75–89. 30531917 10.1038/s41574-018-0122-1

[29] Serin Y, Acar TN. Effect of circadian rhythm on metabolic processes and the regulation of energy balance. Ann Nutr Metab. 2019;74(4):322–30. 31013492 10.1159/000500071

[30] Baron KG, Reid KJ. Circadian misalignment and health. Int Rev Psychiatry. 2014;26(2):139–54. 24892891 10.3109/09540261.2014.911149 PMC4677771

[31] Bass J, Takahashi JS. Circadian integration of metabolism and energetics. Science. 2010;330(6009):1349–54. 21127246 10.1126/science.1195027 PMC3756146

[32] Dibner C. The importance of being rhythmic: living in harmony with your body clocks. Acta Physiol (Oxf). 2020;228(1):e13281. 30980501 10.1111/apha.13281

[33] Mason IC, Qian J, Adler GK, Scheer F. Impact of circadian disruption on glucose metabolism: implications for type 2 diabetes. Diabetologia. 2020;63(3):462–72. 31915891 10.1007/s00125-019-05059-6 PMC7002226

[34] Qian J, Scheer F. Circadian system and glucose metabolism: implications for physiology and disease. Trends Endocrinol Metab. 2016;27(5):282–93. 27079518 10.1016/j.tem.2016.03.005 PMC4842150

[35] Wong PM, Hasler BP, Kamarck TW, Muldoon MF, Manuck SB. Social jetlag, chronotype, and cardiometabolic risk. J Clin Endocrinol Metab. 2015;100(12):4612–20. 26580236 10.1210/jc.2015-2923 PMC4667156

[36] Fonken LK, Nelson RJ. The effects of light at night on circadian clocks and metabolism. Endocr Rev. 2014;35(4):648–70. 24673196 10.1210/er.2013-1051

[37] Wang XS, Armstrong ME, Cairns BJ, Key TJ, Travis RC. Shift work and chronic disease: the epidemiological evidence. Occup Med (Lond). 2011;61(2):78–89. 21355031 10.1093/occmed/kqr001 PMC3045028

[38] Cho Y, Ryu SH, Lee BR, Kim KH, Lee E, Choi J. Effects of artificial light at night on human health: a literature review of observational and experimental studies applied to exposure assessment. Chronobiol Int. 2015;32(9):1294–310. 26375320 10.3109/07420528.2015.1073158

[39] Gao Y, Gan T, Jiang L, Yu L, Tang D, Wang Y, et al. Association between shift work and risk of type 2 diabetes mellitus: a systematic review and dose-response meta-analysis of observational studies. Chronobiol Int. 2020;37(1):29–46. 31684766 10.1080/07420528.2019.1683570

[40] Liu Q, Shi J, Duan P, Liu B, Li T, Wang C, et al. Is shift work associated with a higher risk of overweight or obesity? A systematic review of observational studies with meta-analysis. Int J Epidemiol. 2018;47(6):1956–71. 29850840 10.1093/ije/dyy079

[41] Wolff CA, Esser KA. Exercise timing and circadian rhythms. Curr Opin Physiol. 2019;10:64–9. 31938759 10.1016/j.cophys.2019.04.020 PMC6959205

[42] van der Velde J, Boone SC, Winters-van Eekelen E, Hesselink MKC, Schrauwen-Hinderling VB, Schrauwen P, et al. Timing of physical activity in relation to liver fat content and insulin resistance. Diabetologia. 2023;66(3):461–71. 36316401 10.1007/s00125-022-05813-3 PMC9892088

[43] Poggiogalle E, Jamshed H, Peterson CM. Circadian regulation of glucose, lipid, and energy metabolism in humans. Metabolism. 2018;84:11–27. 29195759 10.1016/j.metabol.2017.11.017 PMC5995632

[44] Scheer FA, Hilton MF, Mantzoros CS, Shea SA. Adverse metabolic and cardiovascular consequences of circadian misalignment. Proc Natl Acad Sci U S A. 2009;106(11):4453–8. 19255424 10.1073/pnas.0808180106 PMC2657421

[45] Morris CJ, Yang JN, Garcia JI, Myers S, Bozzi I, Wang W, et al. Endogenous circadian system and circadian misalignment impact glucose tolerance via separate mechanisms in humans. Proc Natl Acad Sci U S A. 2015;112(17):E2225–34. 25870289 10.1073/pnas.1418955112 PMC4418873

[46] Li W, Chen Z, Ruan W, Yi G, Wang D, Lu Z. A meta-analysis of cohort studies including dose-response relationship between shift work and the risk of diabetes mellitus. Eur J Epidemiol. 2019;34(11):1013–24. 31512118 10.1007/s10654-019-00561-y

[47] Cappuccio FP, D’Elia L, Strazzullo P, Miller MA. Quantity and quality of sleep and incidence of type 2 diabetes: a systematic review and meta-analysis. Diabetes Care. 2010;33(2):414–20. 19910503 10.2337/dc09-1124 PMC2809295

[48] Choi JK, Kim MY, Kim JK, Park JK, Oh SS, Koh SB, et al. Association between short sleep duration and high incidence of metabolic syndrome in midlife women. Tohoku J Exp Med. 2011;225(3):187–93. 22001675 10.1620/tjem.225.187

[49] Bouman EJ, Beulens JWJ, Groeneveld L, de Kruijk RS, Schoonmade LJ, Remmelzwaal S, et al. The association between social jetlag and parameters of metabolic syndrome and type 2 diabetes: a systematic review and meta-analysis. J Sleep Res. 2023;32(3):e13770. 36351658 10.1111/jsr.13770

[50] Gan Y, Yang C, Tong X, Sun H, Cong Y, Yin X, et al. Shift work and diabetes mellitus: a meta-analysis of observational studies. Occup Environ Med. 2015;72(1):72–8. 25030030 10.1136/oemed-2014-102150

[51] Khosravipour M, Khanlari P, Khazaie S, Khosravipour H, Khazaie H. A systematic review and meta-analysis of the association between shift work and metabolic syndrome: the roles of sleep, gender, and type of shift work. Sleep Med Rev. 2021;57:101427. 33556868 10.1016/j.smrv.2021.101427

[52] Wahl S, Engelhardt M, Schaupp P, Lappe C, Ivanov IV. The inner clock-blue light sets the human rhythm. J Biophotonics. 2019;12(12):e201900102. 31433569 10.1002/jbio.201900102 PMC7065627

[53] He JW, Tu ZH, Xiao L, Su T, Tang YX. Effect of restricting bedtime mobile phone use on sleep, arousal, mood, and working memory: a randomized pilot trial. PLoS One. 2020;15(2):e0228756. 32040492 10.1371/journal.pone.0228756 PMC7010281

[54] Kessler K, Hornemann S, Petzke KJ, Kemper M, Kramer A, Pfeiffer AF, et al. The effect of diurnal distribution of carbohydrates and fat on glycaemic control in humans: a randomized controlled trial. Sci Rep. 2017;7:44170. 28272464 10.1038/srep44170 PMC5341154

[55] Pearce KL, Noakes M, Keogh J, Clifton PM. Effect of carbohydrate distribution on postprandial glucose peaks with the use of continuous glucose monitoring in type 2 diabetes. Am J Clin Nutr. 2008;87(3):638–44. 18326602 10.1093/ajcn/87.3.638

[56] Jakubowicz D, Wainstein J, Ahren B, Bar-Dayan Y, Landau Z, Rabinovitz HR, et al. High-energy breakfast with low-energy dinner decreases overall daily hyperglycaemia in type 2 diabetic patients: a randomised clinical trial. Diabetologia. 2015;58(5):912–9. 25724569 10.1007/s00125-015-3524-9

[57] Jakubowicz D, Barnea M, Wainstein J, Froy O. High caloric intake at breakfast vs. dinner differentially influences weight loss of overweight and obese women. Obesity (Silver Spring). 2013;21(12):2504–12. 23512957 10.1002/oby.20460

[58] Morgan LM, Shi JW, Hampton SM, Frost G. Effect of meal timing and glycaemic index on glucose control and insulin secretion in healthy volunteers. Br J Nutr. 2012;108(7):1286–91. 22176632 10.1017/S0007114511006507

[59] Xiao Q, Garaulet M, Scheer F. Meal timing and obesity: interactions with macronutrient intake and chronotype. Int J Obes (Lond). 2019;43(9):1701–11. 30705391 10.1038/s41366-018-0284-x PMC6669101

[60] Garaulet M, Gomez-Abellan P, Alburquerque-Bejar JJ, Lee YC, Ordovas JM, Scheer FA. Timing of food intake predicts weight loss effectiveness. Int J Obes (Lond). 2013;37(4):604–11. 23357955 10.1038/ijo.2012.229 PMC3756673

[61] Bandin C, Scheer FA, Luque AJ, Avila-Gandia V, Zamora S, Madrid JA, et al. Meal timing affects glucose tolerance, substrate oxidation and circadian-related variables: a randomized, crossover trial. Int J Obes (Lond). 2015;39(5):828–33. 25311083 10.1038/ijo.2014.182

[62] Lopez-Minguez J, Gomez-Abellan P, Garaulet M. Timing of breakfast, lunch, and dinner. Effects on Obesity and Metabolic Risk. Nutrients. 2019;11(11):2624. 10.3390/nu11112624 PMC6893547 31684003

[63] Arble DM, Bass J, Laposky AD, Vitaterna MH, Turek FW. Circadian timing of food intake contributes to weight gain. Obesity. 2009;17(11):2100–2. 19730426 10.1038/oby.2009.264 PMC3499064

[64] Bray MS, Ratcliffe WF, Grenett MH, Brewer RA, Gamble KL, Young ME. Quantitative analysis of light-phase restricted feeding reveals metabolic dyssynchrony in mice. Int J Obes (Lond). 2013;37(6):843–52. 22907695 10.1038/ijo.2012.137 PMC3505273

[65] Bray MS, Tsai JY, Villegas-Montoya C, Boland BB, Blasier Z, Egbejimi O, et al. Time-of-day-dependent dietary fat consumption influences multiple cardiometabolic syndrome parameters in mice. Int J Obes (Lond). 2010;34(11):1589–98. 20351731 10.1038/ijo.2010.63 PMC3021134

[66] Bi H, Gan Y, Yang C, Chen Y, Tong X, Lu Z. Breakfast skipping and the risk of type 2 diabetes: a meta-analysis of observational studies. Public Health Nutr. 2015;18(16):3013–9. 25686619 10.1017/S1368980015000257 PMC10271832

[67] Andriessen C, Fealy CE, Veelen A, van Beek SMM, Roumans KHM, Connell NJ, et al. Three weeks of time-restricted eating improves glucose homeostasis in adults with type 2 diabetes but does not improve insulin sensitivity: a randomised crossover trial. Diabetologia. 2022;65(10):1710–20. 35871650 10.1007/s00125-022-05752-z PMC9477920

[68] Chen W, Liu X, Bao L, Yang P, Zhou H. Health effects of the time-restricted eating in adults with obesity: a systematic review and meta-analysis. Front Nutr. 2023;10:1079250. 36875837 10.3389/fnut.2023.1079250 PMC9979543

[69] Kesztyus D, Fuchs M, Cermak P, Kesztyus T. Associations of time-restricted eating with health-related quality of life and sleep in adults: a secondary analysis of two pre-post pilot studies. BMC Nutr. 2020;6(1):76. 33327959 10.1186/s40795-020-00402-2 PMC7745395

[70] Chin SH, Kahathuduwa CN, Binks M. Physical activity and obesity: what we know and what we need to know. Obes Rev. 2016;17(12):1226–44. 27743411 10.1111/obr.12460

[71] Khalafi M, Azali Alamdari K, Symonds ME, Rohani H, Sakhaei MH. A comparison of the impact of exercise training with dietary intervention versus dietary intervention alone on insulin resistance and glucose regulation in individual with overweight or obesity: a systemic review and meta-analysis. Crit Rev Food Sci Nutr. 2023;63(28):9349–63. 35442133 10.1080/10408398.2022.2064424

[72] Malakou E, Linardakis M, Armstrong MEG, Zannidi D, Foster C, Johnson L, et al. The combined effect of promoting the Mediterranean diet and physical activity on metabolic risk factors in adults: a systematic review and meta-analysis of randomised controlled trials. Nutrients. 2018. 10.3390/nu10111577. 30366431 10.3390/nu10111577 PMC6267322

[73] Philippens N, Janssen E, Kremers S, Crutzen R. Short-term outcomes of the digital combined lifestyle intervention CooL-MiGuide: a descriptive case series study. BMC Digital Health. 2025;3(1):70. 10.1186/s12889-023-17501-x PMC10762946 38166961

[74] Mancilla R, Brouwers B, Schrauwen-Hinderling VB, Hesselink MKC, Hoeks J, Schrauwen P. Exercise training elicits superior metabolic effects when performed in the afternoon compared to morning in metabolically compromised humans. Physiol Rep. 2021;8(24):e14669. 33356015 10.14814/phy2.14669 PMC7757369

[75] Allison KC, Hopkins CM, Ruggieri M, Spaeth AM, Ahima RS, Zhang Z, et al. Prolonged, controlled daytime versus delayed eating impacts weight and metabolism. Curr Biol. 2021;31(3):650-7 e3. 33259790 10.1016/j.cub.2020.10.092 PMC7878354

[76] van Rinsum CE, Gerards S, Rutten GM, van de Goor IAM, Kremers SPJ. The coaching on lifestyle (CooL) intervention for obesity, a study protocol for an action-oriented mixed-methods study. BMC Public Health. 2018;18(1):117. 29310640 10.1186/s12889-017-5010-4 PMC5759228

[77] Slebe R, Splinter JJ, Schoonmade LJ, Blondin DP, Campbell DJT, Carpentier AC, et al. The effect of altered sleep timing on glycaemic outcomes: systematic review of human intervention studies. Diabetes Obes Metab. 2025;27(3):1172–83. 39605179 10.1111/dom.16104 PMC11802402

[78] Slebe R, Wenker E, Schoonmade LJ, Bouman EJ, Blondin DP, Campbell DJT, et al. The effect of preprandial versus postprandial physical activity on glycaemia: meta-analysis of human intervention studies. Diabetes Res Clin Pract. 2024;210:111638. 38548105 10.1016/j.diabres.2024.111638

[79] International Organization for Standardization (ISO). ISO 15197:2013 In vitro diagnostic test systems: requirements for blood-glucose monitoring systems for self-testing in managing diabetes mellitus. 2nd ed. Geneva: ISO; 2013. Available from: https://www.iso.org/standard/54976.html.

[80] Ascensia Diabetes Care Netherlands BV. CONTOUR®PLUS BLUE BGMS gebruikershandleidingen. Mijdrecht: Ascensia Diabetes Care Netherlands BV; 2019. Available from: https://www.diabetes.ascensia.nl/siteassets/pdf-files/web90012918_cntrplsblue_bw_ug_r05-24.pdf.

[81] Dunne N, Viggiani MT, Pardo S, Robinson C, Parkes JL. Accuracy evaluation of CONTOUR((R))PLUS compared with four blood glucose monitoring systems. Diabetes Ther. 2015;6(3):377–88. 26169192 10.1007/s13300-015-0121-3 PMC4575307

[82] Stuber JM, Mackenbach JD, de Boer FE, de Bruijn GJ, Gillebaart M, Harbers MC, et al. Correction: Reducing cardiometabolic risk in adults with a low socioeconomic position: protocol of the Supreme Nudge parallel cluster-randomised controlled supermarket trial. Nutr J. 2022;21(1):44. 35764992 10.1186/s12937-022-00795-9 PMC9238004

[83] Elsman EBM, Roorda LD, Crins MHP, Boers M, Terwee CB. Dutch reference values for the patient-reported outcomes measurement information system scale v1.2 - global health (PROMIS-GH). J Patient Rep Outcomes. 2021;5(1):38. 33978855 10.1186/s41687-021-00314-0 PMC8116369

[84] Terwee CB, Roorda LD, de Vet HC, Dekker J, Westhovens R, van Leeuwen J, et al. Dutch-Flemish translation of 17 item banks from the patient-reported outcomes measurement information system (PROMIS). Qual Life Res. 2014;23(6):1733–41. 24402179 10.1007/s11136-013-0611-6

[85] Berendsen M, Boss M, Smits M, Pot GK. Chrono-nutrition and diet quality in adolescents with delayed sleep-wake phase disorder. Nutrients. 2020. 10.3390/nu12020539. 32093078 10.3390/nu12020539 PMC7071432

[86] Phoi YY, Bonham MP, Rogers M, Dorrian J, Coates AM. Content validation of a chrononutrition questionnaire for the general and shift work populations: a Delphi study. Nutrients. 2021. 10.3390/nu13114087. 34836341 10.3390/nu13114087 PMC8620673

[87] Johns MW. A new method for measuring daytime sleepiness: the Epworth sleepiness scale. Sleep. 1991;14(6):540–5. 1798888 10.1093/sleep/14.6.540

[88] van der Pal KC, Koopman ADM, Lakerveld J, van der Heijden AA, Elders PJ, Beulens JW, et al. The association between multiple sleep-related characteristics and the metabolic syndrome in the general population: the New Hoorn study. Sleep Med. 2018;52:51–7. 30278295 10.1016/j.sleep.2018.07.022

[89] Juda M, Vetter C, Roenneberg T. The Munich chronotype questionnaire for shift-workers (MCTQShift). J Biol Rhythms. 2013;28(2):130–40. 23606612 10.1177/0748730412475041

[90] Sekhon M, Cartwright M, Francis JJ. Development of a theory-informed questionnaire to assess the acceptability of healthcare interventions. BMC Health Serv Res. 2022;22(1):279. 35232455 10.1186/s12913-022-07577-3 PMC8887649

[91] Liu D, Huang Y, Huang C, Yang S, Wei X, Zhang P, et al. Calorie restriction with or without time-restricted eating in weight loss. N Engl J Med. 2022;386(16):1495–504. 35443107 10.1056/NEJMoa2114833

[92] van der Heijden AA, Rauh SP, Dekker JM, Beulens JW, Elders P, t Hart LM, et al. The Hoorn Diabetes Care System (DCS) cohort. A prospective cohort of persons with type 2 diabetes treated in primary care in the Netherlands. BMJ Open. 2017;7(5):e015599. 28588112 10.1136/bmjopen-2016-015599 PMC5729999

[93] Castor EDC. Castor Electronic Data Capture 2025 [Available from: https://www.castoredc.com/.

[94] Beintner I, Vollert B, Zarski AC, Bolinski F, Musiat P, Gorlich D, et al. Adherence reporting in randomized controlled trials examining manualized multisession online interventions: systematic review of practices and proposal for reporting standards. J Med Internet Res. 2019;21(8):e14181. 31414664 10.2196/14181 PMC6713038

[95] Stuber JM, van Hoek B, Vos AL, Smit EG, Lakerveld J, Mackenbach JD, et al. Participant recruitment, baseline characteristics and at-home-measurements of cardiometabolic risk markers: insights from the Supreme Nudge parallel cluster-randomised controlled supermarket trial. Trials. 2023;24(1):159. 36864494 10.1186/s13063-023-07157-8 PMC9981252

[96] Fayyaz K, Bataineh MF, Ali HI, Al-Nawaiseh AM, Al-Rifai RH, Shahbaz HM. Validity of measured vs. self-reported weight and height and practical considerations for enhancing reliability in clinical and epidemiological studies: a systematic review. Nutrients. 2024. 10.3390/nu16111704. 38892637 10.3390/nu16111704 PMC11175070

