# Supplementary material for: Metabolic health effects of the timing of lifestyle behaviours in a combined lifestyle intervention in adults who have obesity or are overweight with complications: protocol for a two-armed, cluster-randomized, pragmatic trial—a TIMED study
Source: Trials. 2026 May 20;27:495. doi: 10.1186/s13063-026-09788-z (PMC13366831; doi:10.1186/s13063-026-09788-z)
Supplement: Supplementary file 1 — Additional file 1. [file 13063_2026_9788_MOESM1_ESM.docx]

**Additional file 1. The CooL-Miguide Combined Lifestyle Intervention**

The Combined Lifestyle Intervention (CLI) CooL-MiGuide is the digital adaption of the evidence based ‘Coaching op Leefstijl’ (CooL; translated as Coaching on Lifestyle) program. CooL-MiGuide has been officially implemented and reimbursed in Dutch routine care. This intervention maintains the same design, goals, and evidence-based elements as CooL, with the primary difference being that all sessions are online, rather than in person. This digital format makes CooL-MiGuide suitable for participants that cannot easily join sessions in person due to work, family circumstances, or comorbidities. Participants are therefore slightly younger (49 vs. 52 years nationally) and more highly educated. Because participation requires digital literacy and motivation to engage in an online program, the MiGuide team plans an intake with all potential participants to discuss the motivation and resources before officially starting with CooL-MiGuide. This results in lower drop-out during the intervention (8% vs 26% nationally) ^1-5^.

In addition to the online CooL program, MiGuide provides participants with an online platform and the MiGuide app, the latter originally developed for patients with type 2 diabetes and their health care provider. The connection with CooL makes the app relevant for a broader audience and helps participants of CooL-MiGuide to adhere to the intervention. The app can be tailored to the goals and needs of the participant. Furthermore, the app can be connected to the participants’ medical record ^1-5^.

Preliminary findings show that CooL-MiGuide may be more effective compared to the original CooL, with up to 1.5 times greater improvements in weight, body mass index (BMI), and waist circumference after nine months, although this is only based on 25 participants ^1-5^.

The program consists of a basic program of 6-8 months and maintenance phase of 16-18 months. The basic program consists of:

- One individual intake (60 min.)
  - In this intake the motivation of the participant will be defined, as well as the goals, perspectives, and current and previous lifestyle behaviours.
- Eight group sessions in the basic program (90 min. each) once a month
  - 1. Small steps in behaviour change
  - 2. Physical activity and exercising
  - 3. Healthy diets
  - 4. Get to know yourself, self-regulation strategies, define pitfalls and relapse prevention
  - 5. Stress and relaxation
  - 6. Planning, time management and a healthy lifestyle
  - 7. Healthy sleeping
  - 8. How to maintain the health behaviour changes and keep up with the healthy lifestyle
- Two individual sessions (45 min. each)
  - These give a more individualized perspective and the possibility to discuss personal goals and evaluate the behaviour changes.
- One final individual interview (60 min.)
  - Evaluation of the achieved goals and formulating goals for the future. This interview also serves as an evaluation on whether participation was successful and if the participant should continue with the maintenance phase.

Secondly, a maintenance phase will start for another 16 months. This consists of:

- One intake (60 min.)
- Eight group sessions (90 min. each) once every two months
  - 1. How to maintain a healthy mindset
  - 2. How to stay active and keep exercising
  - 3. Personal balance
  - 4. Healthy diets
  - 5. Healthy leisure time
  - 6. How to feel powerful
  - 7. Know where your chances are
  - 8. How to finish, keep it going on your own, and ask help from others.
- Two individual sessions (45 min. each)
- One individual final interview (60 min.)
  - Evaluation of the achieved goals and formulating goals for the future.

The group sessions will take place in a group of approximately 8-12 participants. A registered lifestyle coach will lead the group sessions, as well as the individual sessions. When needed, participants will be referred to a dietitian or physical therapist to help them with any specific questions or goals.

In addition, participants will get access to the MiGuide app, which is a blended coaching platform that makes personal coaching easier and includes possibilities to track the amount of PA and nutrition intake. In this app, different tasks are defined that can help the participant to reach certain goals. During the program, participants will receive regular notification by email to remind them of the tasks and give them extra information and assignments.

**References**

1. leefstijlinterventies E. Online CooL-programma bij MiGuide n.d. [Available from: <https://leefstijlinterventies.nl/online-cool-programma-bij-miguide/>.

2. MiGuide. MiGuide en CooL-digitaal: een mooie samenwerking 2023 [Available from: <https://miguide.nl/2023/02/16/samenwerking-miguide-cool-digitaal/>.

3. MiGuide. Effectiviteit MiGuide CooL 50% boven landelijk gemiddelde 2023 [Available from: <https://miguide.nl/2023/06/15/tussenmeting-na-9-maanden-miguide-cool/>.

4. MiGuide. Digitale leefstijlinterventie net zo effectief als fysieke interventies? 2023 [Available from: <https://miguide.nl/2023/03/07/digitale-leefstijlinterventie-net-zo-effectief-als-fysieke-interventies/>.

5. Oosterhoff M, Feenstra T, de Wit A. Monitor Gecombineerde Leefstijlinterventie 2023. In: Milieu RvVe, editor. Bilthoven: Rijksinstituut voor Volksgezondheid en Milieu; 2023.
